# CXCL11 Correlates With Antitumor Immunity and an Improved Prognosis in Colon Cancer

**DOI:** 10.3389/fcell.2021.646252

**Published:** 2021-03-11

**Authors:** Yingying Cao, Nanlin Jiao, Tiantian Sun, Yanru Ma, Xinyu Zhang, Haoyan Chen, Jie Hong, Youwei Zhang

**Affiliations:** ^1^Division of Gastroenterology and Hepatology, State Key Laboratory for Oncogenes and Related Genes, Renji Hospital, School of Medicine, Shanghai Institute of Digestive Disease, Shanghai Jiao Tong University, Shanghai, China; ^2^Department of Pathology, Affiliated Yijishan Hospital, Wannan Medical College, Wuhu, China; ^3^Department of Medical Oncology, Xuzhou Central Hospital, Xuzhou Medical University, Xuzhou, China

**Keywords:** CXCL11, colon cancer, immune cell, prognosis, PD-L1, scRNA-seq

## Abstract

The chemokine ligand C-X-C motif chemokine ligand 11 (CXCL11) is involved in the progression of various cancers, but its biological roles in colorectal cancer (CRC) remain confused. Therefore, the prognostic value and underlying mechanism of CXCL11 in CRC were preliminarily evaluated. Three independent datasets were used for mRNA-related analysis: one dataset from the Cancer Genome Atlas (TCGA, *n* = 451) and two single-cell RNA sequencing (scRNA-seq) datasets from Gene Expression Omnibus (GEO): GSE146771 and GSE132465. In addition, a colon adenocarcinoma (COAD) patient cohort (the Yijishan Hospital cohort, YJSHC, *n* = 108) was utilized for analysis of cell infiltration by immunohistochemistry. We determined the distribution of CXCL11 in tumor tissue across all TCGA cancers and found that CXCL11 expression was significantly upregulated in both COAD and rectal adenocarcinoma (READ). However, the upregulation of CXCL11 mRNA was associated with a better prognosis in COAD, but not in READ. Within the YJSHC, the patients with a high abundance of intratumoral CXCL11^+^ cells had prolonged survival (*p* = 0.001). Furthermore, we found that the high CXCL11 expression group had a higher proportion of antitumor immune cells, and a lower proportion of protumor immune cells. Additionally, we discovered the changes of gene expression and enriched immune pathway network mediated by CXCL11. Interestingly, both cytotoxic genes (IFNG, GZMA, GZMB, GZMK, GZMM, and PRF1) and immunosuppressive molecules, including PD-L1, were positively correlated with CXCL11 expression. CXCL11, which promoted antitumor immunity to benefit survival, was identified as an independent prognostic biomarker in patients with COAD.

## Introduction

Colorectal cancer (CRC) is a commonly occurring cancer and remains the third leading cause of cancer-related death worldwide (Arnold et al., 2017). The CRC incidence in China has increased over the past two decades, but no significant improvements in prognosis have been achieved (Chen et al., 2016). Therefore, the molecular mechanisms underlying CRC development should be further explored, and novel biomarkers for prognostic evaluation should be identified. Evidence suggests the involvement of the tumor microenvironment (TME), especially immune cells, in the development of CRC in addition to genetic mutations (Cancer Genome Atlas Network., 2012; Ge et al., 2019). The distinct roles of antitumor immunity are dependent on cytokine-cytokine interactions, which constitute the cytokine networks that normally maintain intestinal homeostasis (West et al., 2015). As a family of small signaling cytokines, chemokines are a very important part of the cross talk between tumor cells and the microenvironment, and these molecules interact with their receptors to regulate the leukocyte infiltration, tumor related angiogenesis, host immune response activation and tumor cell proliferation (Cabrero-de, Las Heras and Martínez-Balibrea, 2018).

The chemokine ligand C-X-C motif chemokine ligand 11 (CXCL11), also known as IFN-inducible T cell α chemoattractant (I-TAC), mediates recruitment of T cells, natural killer (NK) cells and monocytes/macrophages at sites of infection, predominantly through the cognate G-protein coupled receptor CXCR3, like CXCL9 and CXCL10 (Colvin et al., 2004; Karin, 2020). This signaling axis has been implicated in several physiological activities, including immune cell migration, differentiation, and activation (Tokunaga et al., 2018). Data from The Cancer Genome Atlas (TCGA) and Gene Expression Omnibus (GEO) show that CXCL11 expression is upregulated in colon cancer tissue compared with healthy tissue, and that a high level of CXCL11 is correlated with prolonged survival (Liu et al., 2020). CXCL11-dependent therapy may be a potential approach for cancer treatment (Liu et al., 2016; Moon et al., 2018). However, the expression level of CXCR3 in clinical cancer samples is associated with metastatic potential and patients prognosis (Zhang Y. et al., 2018), and the affinity of CXCL11 for CXCR3 is the highest of the three selective ligands (Cole et al., 1998). From this perspective, CXCL11 may contribute to tumor progression. A study on BALB/c mice bearing CT26.WT cell xenografts showed enhanced tumor growth and invasiveness after peritumoral application of CXCL11 to the TME (Rupertus et al., 2014). It has also been shown that repression of CXCL11 inhibits CRC cell growth and epithelial-mesenchymal transition (EMT) (Gao et al., 2018). In addition, the secretion of CXCL11 by neuroendocrine-like cells can recruit tumor-associated macrophages (TAMs) to infiltrate tumor tissues, which enhances the proliferation and invasion of CRC cells and leads to poor prognosis (Zeng et al., 2016). Therefore, the role of CXCL11 in CRC pathogenesis is still unclear.

In the present study, we first discovered that CXCL11 mRNA expression was upregulated in both colon adenocarcinoma (COAD) and rectal adenocarcinoma (READ) samples in the TCGA database, but only the upregulation in COAD had prognostic value. Therefore, we focused on COAD in follow-up research. CXCL11 protein expression was confirmed in our cohort by immunohistochemistry (IHC), and the effect of CXCL11 on the tumor immune microenvironment was also evaluated.

## Materials and Methods

### Patient Samples

Three independent datasets were included in this study: one dataset from the TCGA (*n* = 451) and two single-cell RNA sequencing (scRNA-seq) datasets from GEO (GSE146771 and GSE132465). In addition, the patient cohort YJSHC (the Yijishan Hospital cohort, *n* = 108) was also involved. The characteristics of patients in the TCGA were downloaded from http://www.cbioportal.org on 18 April 2020. The clinical information of enrolled patients in the TCGA and YJSHC in this study are provided in Supplementary Table 1 and Supplementary Table 2. The YJSHC consisted of patients with COAD from Yijishan Hospital affiliated with Wannan Medical College (Wuhu, China), who underwent surgery between August 2011 and December 2014. All of the research was carried out in accordance with the Declaration of Helsinki and approved by the ethics committee of Wannan Medical College, Yijishan Hospital. Additionally, informed consent was obtained from every patient.

### Immunohistochemistry (IHC)

We collected formalin-fixed, paraffin-embedded surgical specimens, which were used for tissue microarray construction and subsequent immunohistochemistry studies. First, tissue microarray sections were rehydrated, and treated with hydrogen peroxide and then heat-mediated antigen retrieval by microwaving with sodium citrate. Next, the slides were incubated with the indicated antibodies obtained from Abcam (Cambridgeshire, United Kingdom) in a humidified box at 4°C overnight. The details for the four antibodies used in this study are provided in Supplementary Table 3. Next, the color reaction was realized by a DAB substrate kit and nucleus counterstaining was performed with hematoxylin. The level of protein expression was assessed based on the intensity of staining and the extent of staining at 200 × under a microscope. To determine the extent of staining, a quantity score (0–4) denoted 0, 0%; 1, 1–10%; 2, 11–50%; 3, 51–80% and 4, 81–100% of positive cells, was used. The staining intensity was divided into three grades: weak, moderate, and strong staining. It should be noted that the corresponding intensity scores ranged from 1 to 3. The final IHC score was calculated by multiplying the quantity and intensity scores.

### Immune Cell Infiltration Analysis

The relative abundance of the tumor infiltrating lymphocytes (TILs) in COAD tissues with different CXCL11 mRNA expression statuses was calculated by CIBERSORT^1^ (Newman et al., 2015) and TISIDB^2^ (Ru et al., 2019). For each cancer type, GSVA (gene set variation analysis) was adopted to infer the relative proportion of various TILs based on the immune-related gene expression profile of 28 TIL types from Charoentong’s study (Charoentong et al., 2017). The correlation between CXCL11 and TILs was measured by Spearman’s test. In this study, we also investigated the differential expression of CXCL11 between tumor and adjacent normal tissues across all TCGA tumors in TIMER^3^ (Li et al., 2017).

### Differential Expression Analysis

The R package “limma” was used for differential expression analysis. Differentially expressed genes between CXCL11-high and CXCL11-low tumor samples were defined as the up-regulated or down-regulated genes with a fold change ≥ 2 and false discovery rate (FDR) *P* < 0.05.

### Gene Set Enrichment Analysis (GSEA)

The significantly enriched pathways in CXCL11-high tumor samples were identified by gene set enrichment analysis (GSEA) performed by “MSigDB” (Molecular Signatures Database, version 6.0). Additionally, the enrichment score was assessed by determining the expression of a gene set. When the majority of a gene set had elevated expression, we considered the gene set to be a “enriched” gene set.

### Single-Cell Transcriptomic Analysis

External single-cell transcriptomic data of COAD patients were retrieved from GEO (GSE146771 and GSE132465). Single-cell data analysis was performed using scanpy v.1.4^4^. Since single-cell technologies currently capture only a portion of the transcripts in any cell, we generated “pseudobulks” as technical replicates by summing the raw UMI counts for each gene from the same patient.

### Statistical Analysis

In this study, R and corresponding R packages were utilized for statistical analysis. We used the Cutoff Finder platform^5^ to automatically determine the cutoff points (Budczies et al., 2012). The results were all displayed as the mean ± SD. Statistical significance was defined by a two-tailed *P* < 0.05. Student’s *t*-test was adopted for the analysis of continuous variables. Spearman’s correlation was performed to analyze correlations. We performed survival analysis with Kaplan-Meier method, and survival was compared among multiple groups with the log-rank test.

## Results

### Aberrant Expression and Prognostic Value of CXCL11 in Colon Cancer

**FIGURE 1:**
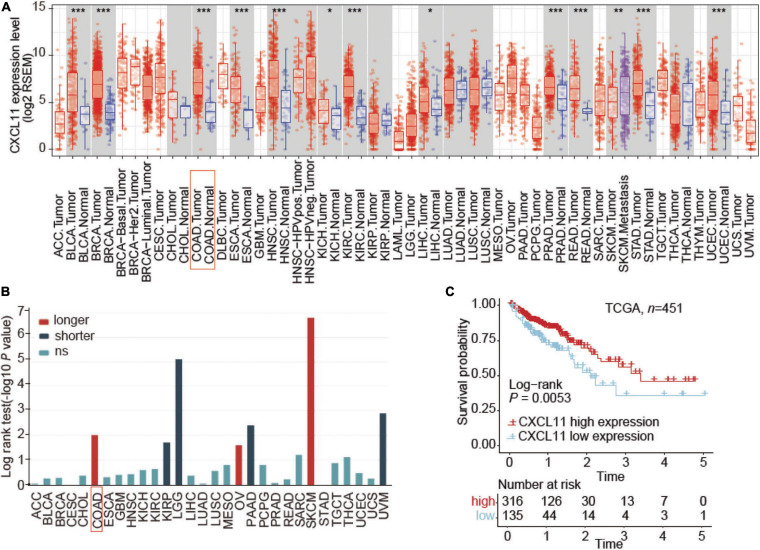
Aberrant expression and prognostic value of CXCL11 in the TCGA. **(A)** The relative level of CXCL11 mRNA expression in all TCGA tumors. **(B)** Correlation between CXCL11 and clinical outcome in all TCGA tumors, *P < 0.05, **P < 0.01, and ***P < 0.001. **(C)** Survival curves of TCGA data stratified by CXCL11 mRNA expression. ACC, adrenocortical carcinoma; BLCA, bladder urothelial carcinoma; BRCA, breast carcinoma; CESC, cervical squamous carcinoma; CHOL, cholangiocarcinoma; COAD, colon adenocarcinoma; ESCA, esophageal carcinoma; GBM, glioblastoma multiforme; HNSC, head and neck squamous cell carcinoma; KICH, kidney chromophobe; KIRC, kidney renal clear cell carcinoma; KIRP, kidney renal papillary cell carcinoma; LGG, lower grade glioma; LIHC, liver hepatocellular carcinoma; LUAD, lung adenocarcinoma; LUSC, lung squamous cell carcinoma; MESO, mesothelioma; OV, ovarian serous adenocarcinoma; PAAD, pancreatic ductal adenocarcinoma; PCPG, paraganglioma and pheochromocytoma; PRAD, prostate adenocarcinoma; READ, rectum adenocarcinoma; SARC, sarcoma; SKCM, skin cutaneous melanoma; STAD, stomach adenocarcinoma; TGCT, testicular germ cell cancer; THCA, thyroid papillary carcinoma; UCEC, uterine corpus endometrioid carcinoma; UCS, uterine corpus squamous carcinoma; UVM, uveal melanoma.

First, we evaluated the distribution of CXCL11 in tumor tissue across all TCGA cancers in TIMER and found that CXCL11 expression was significantly upregulated in the majority of tumors, including COAD and READ (Figure 1A). Next, TISIDB was utilized to examine the association between CXCL11 and clinical outcome across all TCGA tumors, and we found that COAD patients but not READ patients with high mRNA expression of CXCL11 had a better prognosis, instead of READ (Figure 1B). Therefore, we focused on the prognostic significance of CXCL11 in COAD, and we downloaded a dataset from the TCGA (*n* = 451). The results revealed that high CXCL11 mRNA expression was associated with prolonged survival (*P* = 0.0053, Figure 1C).

### Association of CXCL11 With the Tumor Immune Microenvironment

**FIGURE 2:**
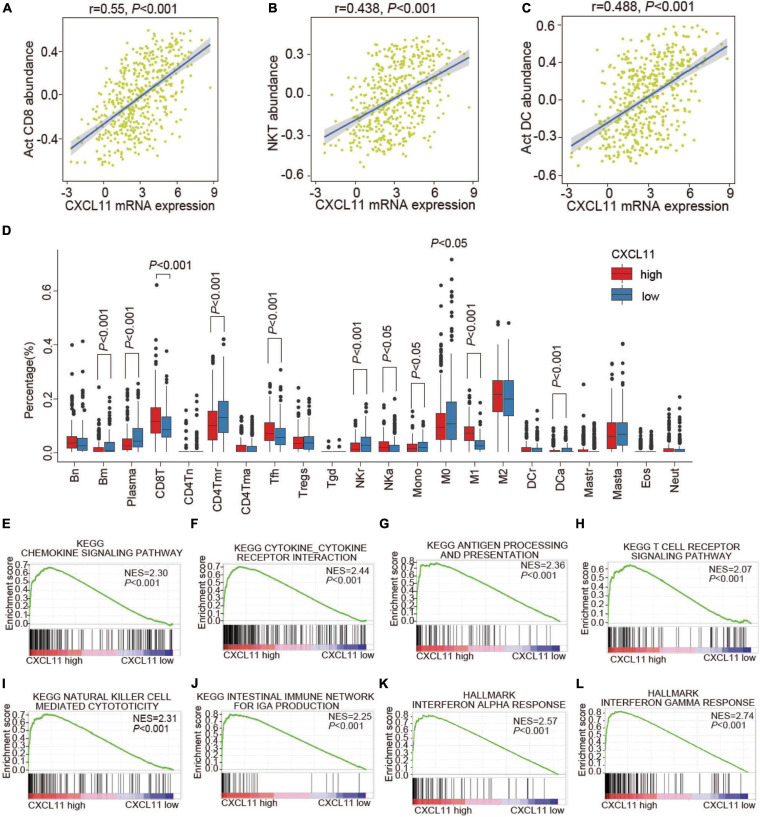
The infiltration of various TILs and enriched biological pathways compared between the high and low CXCL11 groups. **(A–C)** The correlations between CXCL11 and the abundances of Act CD8 (activated CD8^+^ T cells) **(A)**, NKT (natural killer T cells) **(B)**, Act DC (activated dendritic cells) **(C)** in TISIDB. **(D)** The infiltration of various TILs identified by CIBERSORT in the high and low CXCL11 groups in the TCGA. Bn = naive B cells; Bm = memory B cells; Plasma = plasma cells; CD8T = CD8^+^ T cells; CD4Tn = naive CD4^+^T cells; CD4Tmr = resting memory CD4^+^T cells; CD4Tma = activated memory CD4^+^T cells; Tfh = follicular helper T cells; Tregs = regulatory T cells; Tgd = γδT cells; NKr = resting natural killer cells; NKa = activated natural killer cells; Mono = monocytes; M0 = M0 macrophages; M1 = M1 macrophages; M2 = M2 macrophages; DCr = resting dendritic cells; DCa = activated dendritic cells; Mastr = resting mast cells; Masta = activated mast cells; Eos = eosinophils; Neut = neutrophils. **(E–L)** Gene set enrichment analysis (GSEA) revealed the enriched biological pathways in the high CXCL11 group. NES, normalized enrichment score.

To understand the underlying mechanism of CXCL11 in COAD, we investigated the associations of CXCL11 with the tumor immune microenvironment. TISIDB was employed to investigate which kinds of TILs might be regulated by CXCL11 across all TCGA tumors (Supplementary Figure 1). We found that CXCL11 was positively correlated with Act CD8 (activated CD8^+^ T cells; *r* = 0.55, *P* < 0.001), NKT (natural killer T cells; *r* = 0.438, *P* < 0.001), and Act DC (activated dendritic cells; *r* = 0.488, *P* < 0.001) in COAD, as shown in Figures 2A–C.

Furthermore, the differential status of the tumor immune microenvironment in different groups of COAD patients determined by CXCL11 mRNA expression patterns was validated by CIBERSORT (Figure 2D). Consistently, the high CXCL11 mRNA expression group had a higher proportion of antitumor immune cells, such as: CD8T (CD8^+^ T cells, *P* < 0.001), NKa (activated natural killer cells, *P* < 0.05). Additionally, the high CXCL11 mRNA expression group had a lower proportion of protumor immune cells, such as: M0 (M0 macrophages, *P* < 0.05), NKr (resting natural killer cells, *P* < 0.001), and Mono (monocytes, *P* < 0.05). The results suggested that CXCL11 was associated with antitumor immunity in COAD, which partially explained the association of CXCL11 with a better prognosis.

### Differentially Expressed Genes and Enriched Biological Processes in the High and Low CXCL11 Metagene Expression Groups

As CXCL11 was correlated with antitumor immunity, we aimed to identify the differentially expressed genes and enriched biological pathways mediated by CXCL11 in the tumor immune microenvironment. First, we performed GSEA and identified that the biological pathways enriched in CXCL11-high tumor samples were the chemokine signaling pathway (NES = 2.3, *P* < 0.001), cytokine-cytokine receptor interaction (NES = 2.44, *P* < 0.001), antigen processing and presentation (NES = 2.36, *P* < 0.001), T cell receptor signaling pathway (NES = 2.07, *P* < 0.001), natural killer cell mediated cytotoxicity (NES = 2.31, *p* < 0.001), intestinal immune network for IgA production (NES = 2.25, *P* < 0.001), interferon alpha response (NES = 2.57, *P* < 0.001), and interferon gamma response (NES = 2.74, *P* < 0.001), which are known to contribute to the antitumor immunity, as shown in Figures 2E–L.

**FIGURE 3:**
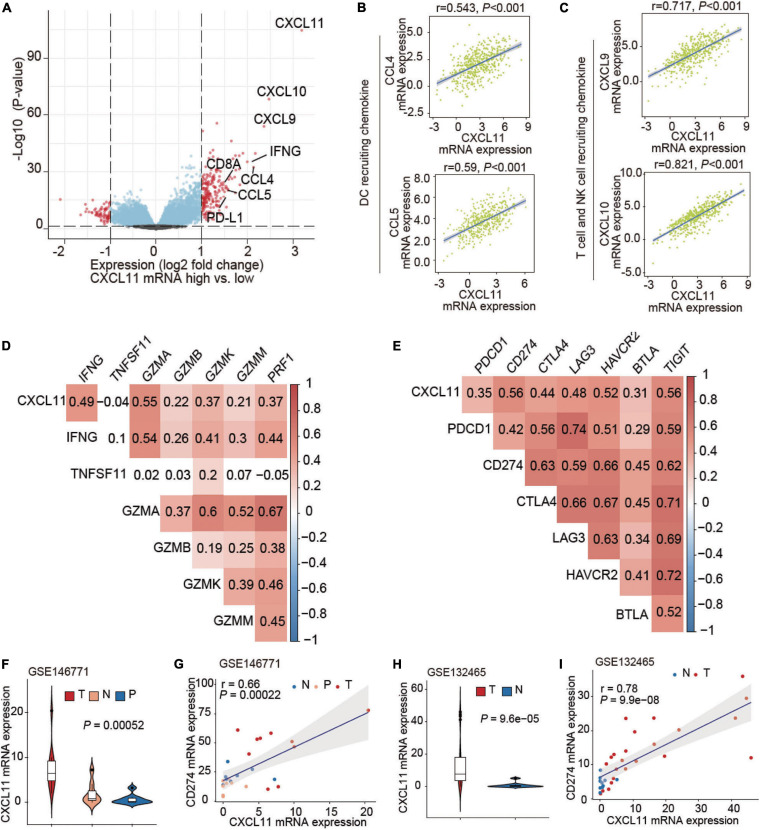
Associations of CXCL11 with immune-related genes. **(A)** Volcano plot showing the differentially expressed genes between the high and low CXCL11 groups. **(B,C)** Correlations between CXCL11 and the indicated transcripts of DC and T cell-recruiting chemokines. **(D,E)** Correlations between CXCL11 and cytotoxic molecules **(D)**, or immunosuppressive genes **(E)**. **(F)** The differential expression of CXCL11 in N (normal tissue), P (para-carcinoma tissue), and C (cancer tissue) in the single-cell RNA-seq dataset GSE146771. **(G)** Spearman’s correlation analysis of CXCL11 and PD-L1 in the single-cell RNA-seq datasets GSE146771. **(H)** The differential expression of CXCL11 in N (normal tissue), P (para-carcinoma tissue) and C (cancer tissue) in the single-cell RNA-seq dataset GSE132465. **(I)** Spearman’s correlation analysis of CXCL11 and PD-L1 in the single-cell RNA-seq datasets GSE132465.

We then studied genetic alterations and found that the differentially expressed genes in tumors in the high CXCL11 mRNA expression group included various immune-activated genes. A volcano plot is shown in Figure 3A, and we observed that the CXCL11- high group was enriched in CXCL9, CXCL10, CCL4, CCL5, IFNG, CD8A, and PD-L1. As shown in Figures 3B,C, CXCL11 was positively correlated with a gene set associated with DC-, NK cell and T cell recruiting CCL4 (*r* = 0.543, *P* < 0.001), CCL5 (*r* = 0.59, *P* < 0.001), CXCL9 (*r* = 0.717, *P* < 0.001), and CXCL10 (*r* = 0.821, *P* < 0.001). In some ways, CXCL11 was found to contribute to the antitumor immune microenvironment in COAD.

Cytotoxic genes including IFNG, GZMA, GZMB, GZMK, GZMM, and PRF1 were positively correlated with CXCL11 mRNA expression (Spearman’s q = 0.49, 0.55, 0.22, 0.37, 0.21, and 0.37; all *P*-values are < 0.001), as shown in Figure 3D. Interestingly, several immunosuppressive molecules including PDCD1, PD-L1, and CTLA4 were positively correlated with CXCL11 mRNA expression (Spearman’s q = 0.35, 0.56, and 0.44; *P* < 0.001, *P* < 0.001, and *P* < 0.001, respectively), as shown in Figure 3E. Furthermore, we found upregulation of CXCL11 expression in tumor tissue compared with adjacent normal tissue (Figure 3F) in the COAD single-cell RNA-seq datasets GSE146771, and CXCL11 expression was positively correlated with PD-L1 (*r* = 0.66, *P* < 0.001; Figure 3G). In addition, the UMAP plots showing the expression levels of CXCL11 and PD-L1 in different clusters of GSE146771 were in Supplementary Figures 2A–C. We also found the upregulation of CXCL11 expression in tumor tissue compared with adjacent normal tissue (Figure 3H) in the COAD single-cell RNA-seq datasets GSE132465, and CXCL11 expression was positively correlated with PD-L1 (*r* = 0.78, *P* < 0.001; Figure 3I).

### Differential Expression and Prognostic Value of CXCL11^+^ Cells in YJSHC

**FIGURE 4:**
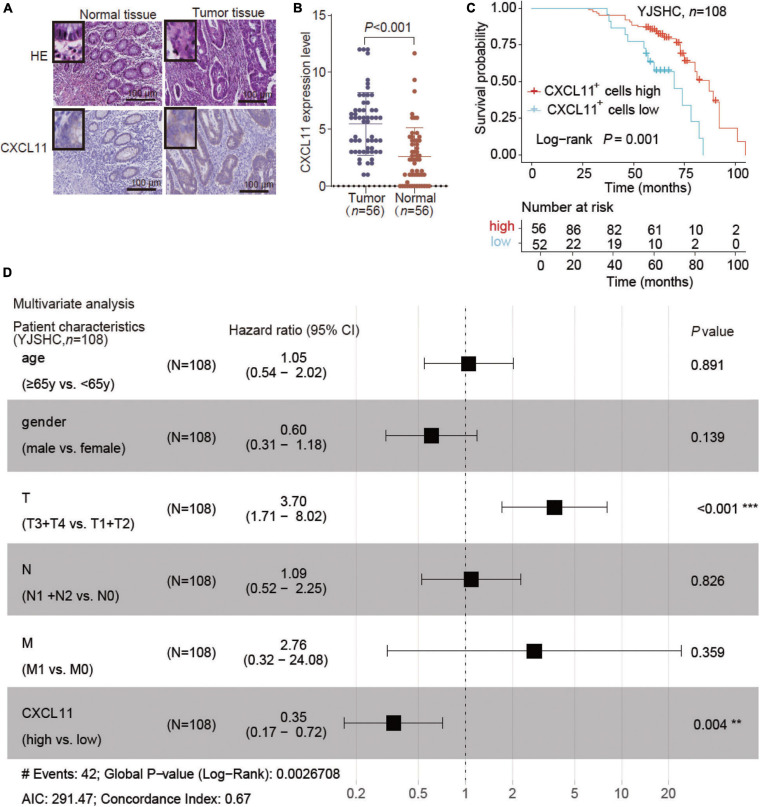
Expression of CXCL11 and effect of CXCL11^+^ cells on the survival of patients in the Yijishan Hospital cohort (YJSHC). **(A,B)** Comparison of CXCL11^+^ cells between tumor and adjacent normal tissues in the YJSHC. **(C)** Overall survival curves of patients in the YJSHC stratified by intratumoral CXCL11^+^ cells levels. **(D)** Multivariate Cox analysis of CXCL11^+^ cell infiltration and clinicopathological variables. **P < 0.01, and ***P < 0.001.

To further validate the effect of CXCL11, we enrolled the COAD patient cohort from Yijishan Hospital (YJSHC). First, we verified the upregulated protein expression of CXCL11 in tumor tissue compared to adjacent normal tissue (Figures 4A,B) by IHC staining. Next, the patients were divided into two groups based on their CXCL11 expression and the respective clinical outcomes were analyzed. We found that the patients within the YJSHC with a high abundance of intratumoral CXCL11^+^ cells had better OS (overall survival, *P* = 0.001) within the YJSHC, as shown in Figure 4C. Additionally, multiple Cox regression analysis was used to verify that the prognostic value of intratumoral CXCL11^+^ cell infiltration kept significant after adjusting for confounders (*HR* = 0.35; 95% CI 0.17–0.72; *P* = 0.004; Figure 4D). It was confirmed that CXCL11 could be an independent prognostic biomarker.

### Validating the Associations of CXCL11^+^ Cells With TILs and PD-L1

**FIGURE 5:**
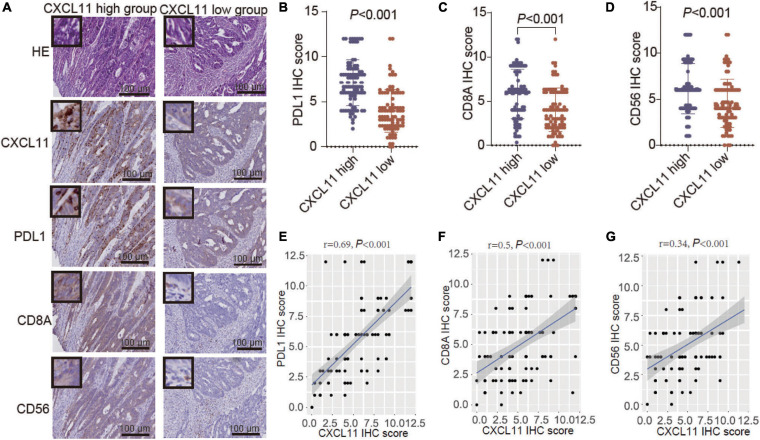
Verification of TILs in different groups, which were generated according to CXCL11^+^ cell infiltration levels, by immunohistochemistry (IHC). **(A–D)** Comparison of the infiltration of PD-L1^+^, CD8A^+^, and CD56^+^ cells between the high and low intratumoral CXCL11^+^ cell groups in the YJSHC. **(E–G)** Correlation of CXCL11 with PD-L1, CD8, and CD56 in the YJSHC.

Further analysis of the YJSHC was conducted to validate the immunomodulatory effect of CXCL11^+^ cell infiltration. As shown in serial sections (Figure 5A), tumor with a high abundance of CXCL11^+^ cell infiltration tended to have a high abundance of intratumoral CD8^+^ T cell and CD56^+^ NK cell infiltration, which was associated with antitumor immunity. In addition, a high abundance of CXCL11^+^ cell infiltration was associated with a high abundance of intratumoral PD-L1^+^ cells. Accordingly, the expression of PD-L1, CD8A, and CD56 was higher in the CXCL11-high group than in the CXCL11-low group (Figures 5B–D), and the positive correlations between the expression of CXCL11 and PD-L1, CD8A, or CD56 (*r* = 0.62, *P* < 0.001; *r* = 0.34, *P* < 0.001; *r* = 0.32, *P* < 0.001, respectively; Figures 5E–G) were significant within the YJSHC.

## Discussion

COAD and READ are regarded in all fields of research and clinical practice as a single tumor entity termed CRC. In fact, obvious differences exist in molecular carcinogenesis, pathology, surgical procedures and multimodal treatment, and COAD is not the same as READ (Paschke et al., 2018). In this study, we found upregulation of CXCL11 expression in COAD tumor tissues compared with normal tissues in the TCGA, GEO single-cell RNA-seq datasets and YJSHC cohort, and that elevated CXCL11 had independent prognostic value in COAD. The results confirm that upregulation of CXCL11 expression plays a defensive role in COAD development.

CXCL11 is a major chemoattractant for effector T cells (Cole et al., 1998). There have been already several evidences to support our findings (Li et al., 2019; Liu et al., 2020). In a mesothelioma mouse model, a tumor-selective oncolytic vaccinia virus expressing CXCL11 reportedly enhanced tumor infiltrating CTLs and NK cells, but not CD4^+^ T cells, and prolonged survival (Liu et al., 2016). Gao et al. (2019) found that docetaxel induced CD8^+^ T cell recruitment into the tumor microenvironment by enhancing the secretion of CXCL11, thus improving antitumor efficacy, and that increased CXCL11 expression was positively correlated with prolonged OS in lung cancer patients. However, previous studies also identified a role for CXCL11 in the regulation of the oncogenic process in various types of human cancers, including colorectal cancer (Rupertus et al., 2014; Zeng et al., 2016). A high level of CXCL11 is associated with worse TNM staging in patients with pancreatic cancer (PC), enhancing the proliferation and metastasis of PC cells (Ge et al., 2020). In addition, CXCL11 participants in the maintenance of the stem cell-like properties of hepatocellular carcinoma (HCC) α2δ1^+^ tumor initiating cells (TICs), and promotes self-renewal and tumorigenicity in α2δ1^+^ liver TICs through CXCR3/ERK1/2 signaling (Zhao et al., 2013; Zhang et al., 2019). The paradox of the opposing effects of CXCL11 expression may be due to the context-specific manner by which CXCL11 regulates cellular processes through different regulatory networks.

Tumor-infiltrating immune cells are closely related to tumorigenesis, angiogenesis and tumor cell growth and metastasis, which could in turn regulate the quantity and differentiation of immune cells (Di Caro et al., 2013). Evidence indicates that tumor progression may result from the escape of cancer cells from host immunosurveillance (Spinner et al., 2019). Therefore, clarifying the infiltrating immune cells in the TME may help elucidate the underlying mechanism involving CXCL11 in COAD. We found that the high CXCL11 expression group had a higher proportion of antitumor immune cells, and a lower proportion of protumor immune cells. Furthermore, we discovered the differentially expressed genes and enriched immune pathway network mediated by CXCL11. We found that CXCL11 was positively correlated with CCL4, CCL5, CXCL9, and CXCL10 in COAD, which are associated with DC, NK, and T cell recruitment and play important roles in inhibiting tumor growth and improving prognosis (Böttcher et al., 2018; Cabrero-de, Las Heras and Martínez-Balibrea, 2018). Accordingly, we found positive correlations between CXCL11 and cytotoxic genes (IFNG, GZMA, GZMB, GZMK, and GZMM), which were validated to enhance the cytotoxic function by other immunocytes and contribute to the immunostimulatory microenvironment (Garzón-Tituaña et al., 2020). These data indicate that CXCL11 promotes antitumor immunity by mediating the infiltration of immunocytes. This mechanism may be different from the classic signaling pathway triggered by the binding of CXCL11 to its receptor CXCR3 or CXCR7 (Puchert et al., 2020).

Interestingly, our study found that CXCL11 expression was positively correlated with PD-L1 in both the TCGA and the YJSHC, which was also verified in the COAD single-cell RNA-seq datasets GSE146771 and GSE132465. This discovery was consistent with the finding that PD-L1 is upregulated after treatment with CXCL11, accompanied by activation of STAT3 and Akt in gastric cancer (Zhang C. et al., 2018). Considering that other immunosuppressive molecules, including PDCD1, and CTLA4, were positively correlated with CXCL11 mRNA expression, the mechanism underlying the correlation between CXCL11 and the immune contexture needs further investigations. Several studies have shown that massive intratumoral CD8^+^ T cell infiltration is able to alleviate the resistance to immune checkpoint inhibitors (ICIs) therapy in some way (Tumeh et al., 2014; Hegde et al., 2016). Nevertheless, PD-L1 blockade combined with an oncolytic vaccinia virus expressing CXCL11 in murine tumor models was shown to significantly reduce tumor burden and improve prognosis (Jonas, 2017). It’s promising to validate whether CXCL11 can predict the response to ICIs in COAD.

## Conclusion

Our study identified that elevated CXCL11 was an independent prognostic biomarker in patients with COAD, that promoted anti-tumor immunity to prolong survival. In addition, high expression of PD-L1 induced by CXCL11 in COAD perhaps improve the therapeutic response of patients receiving ICI treatment. Thus, our findings can provide novel insights to assist clinicians in choosing appropriate measures for their patients and improve the long-term outcome of COAD.

## Data Availability Statement

The datasets generated for this study can be found in the online repositories. The names of the repository/repositories and accession number(s) can be found in the article/Supplementary Material.

## Ethics Statement

The studies involving human participants were reviewed and approved by the Ethics Committee of Yijishan Hospital, Wannan Medical College. The patients/participants provided their written informed consent to participate in this study.

## Author Contributions

YC, TS, and YM performed the experiments. YZ, NJ, and XZ collected the patient samples. YZ, YC, and HC performed the data analysis work and wrote the manuscript. HC and JH designed the study and assisted in writing the manuscript. All authors read and approved the final manuscript.

## Conflict of Interest

The authors declare that the research was conducted in the absence of any commercial or financial relationships that could be construed as a potential conflict of interest.

## Footnotes

1 https://cibersort.stanford.edu/

2 http://cis.hku.hk/TISIDB/index.php

3 https://cistrome.shinyapps.io/timer/

4 https://scanpy.readthedocs.io/en/stable/

5 http://molpath.charite.de/cutoff

## Supplementary Material

Supplementary Figure 1 Tumor infiltrating lymphocytes (TILs) regulated by CXCL11 across all TCGA tumors.

Supplementary Figure 2 UMAP plots showing expression levels of selected genes in different clusters of GSE146771.

Supplementary Table 1 Enrolled patient characteristics (TCGA, *n* = 451; YJSHC, *n* = 108).

Supplementary Table 2 Clinical information of 108 cases of colon cancer patients.

Supplementary Table 3 Immunohistochemistry (IHC) antibodies.
