# Supplementary figures and images for: CXCL11 Correlates With Antitumor Immunity and an Improved Prognosis in Colon Cancer
Source: Front Cell Dev Biol. 2021 Mar 11;9:646252. doi: 10.3389/fcell.2021.646252 (PMC7991085; doi:10.3389/fcell.2021.646252)

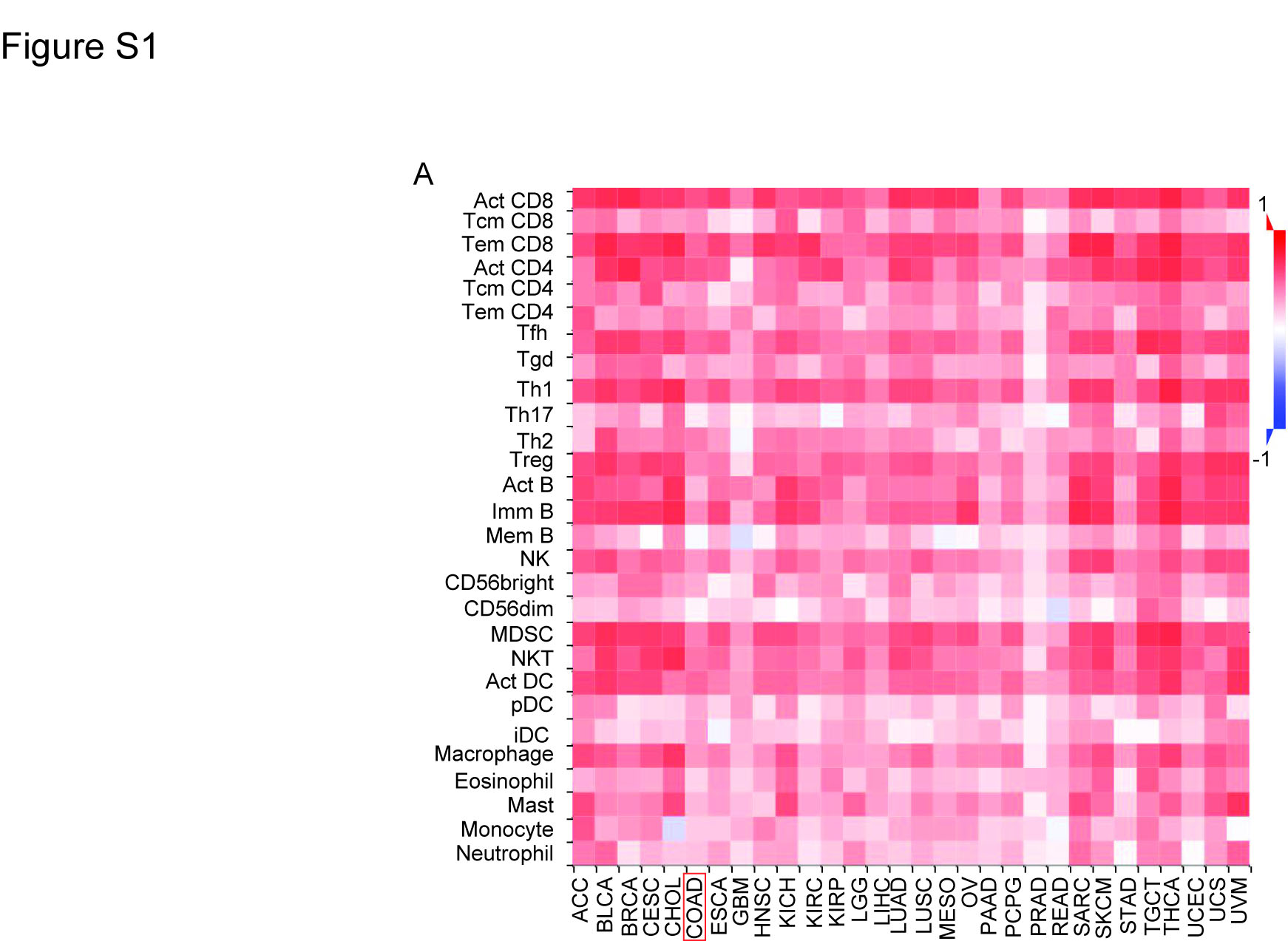

Supplement: Supplementary Figure 1 — Tumor infiltrating lymphocytes (TILs) regulated by CXCL11 across all TCGA tumors. [file Image_1.JPEG]

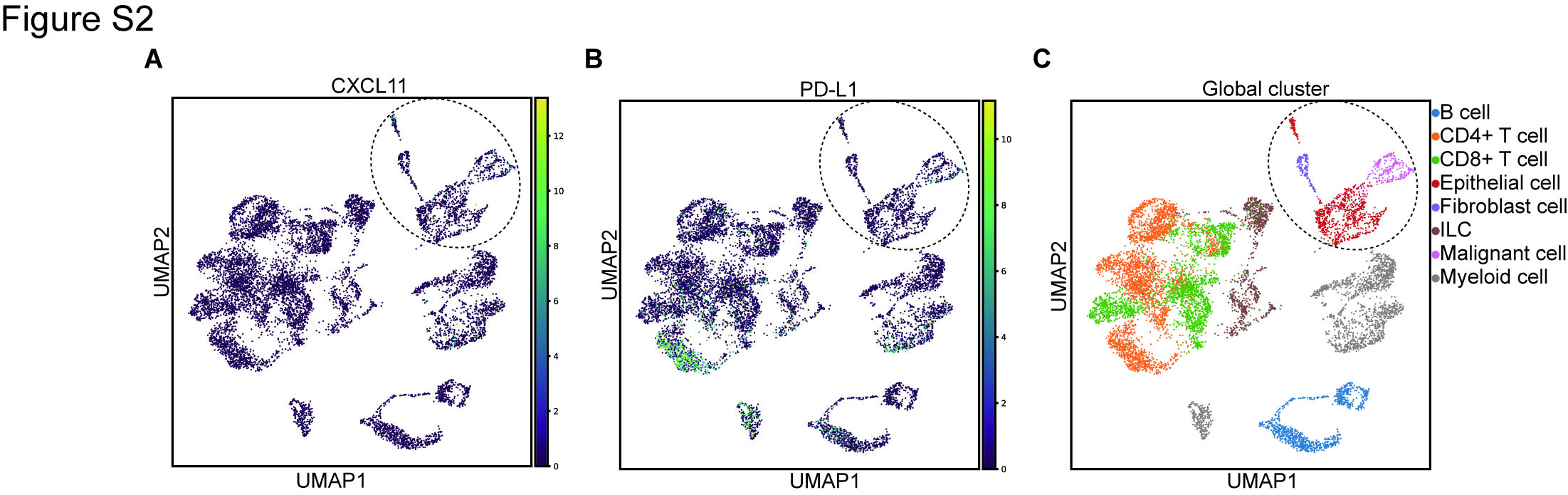

Supplement: Supplementary Figure 2 — UMAP plots showing expression levels of selected genes in different clusters of GSE146771. [file Image_2.JPEG]
