# Supplementary material for: CXCL11 Correlates With Antitumor Immunity and an Improved Prognosis in Colon Cancer
Source: Front Cell Dev Biol. 2021 Mar 11;9:646252. doi: 10.3389/fcell.2021.646252 (PMC7991085; doi:10.3389/fcell.2021.646252)
Supplement: Supplementary Table 1 — Enrolled patient characteristics (TCGA, n = 451; YJSHC, n = 108). [file Table_1.DOCX]

Supplementary Material

| **Supplementary Table 1.** **Enrolled patient characteristics (TCGA, *n*=451; YJSHC， *n*=108).** | | | | | | | | | | | |
| --- | --- | --- | --- | --- | --- | --- | --- | --- | --- | --- | --- |
| **Patient characteristics** |  | **TCGA** | | |  |  | **YJSHC** | | |  |  |
|  |  | ***n*** |  | % |  |  | ***n*** |  | % |  |  |
| Total |  | 451 |  | 100 |  |  | 108 |  | 100 |  |  |
| CXCL11 mRNA expression |  |  |  |  |  |  |  |  |  |  |  |
| Low |  | 135 |  | 29.9 |  |  |  |  |  |  |  |
| High |  | 316 |  | 70.1 |  |  |  |  |  |  |  |
| CXCL11^+^ cells (intratumor) |  |  |  |  |  |  |  |  |  |  |  |
| Low |  |  |  |  |  |  | 52 |  | 44.6 |  |  |
| High |  |  |  |  |  |  | 56 |  | 55.4 |  |  |
| Age |  |  |  |  |  |  |  |  |  |  |  |
| <65year |  | 172 |  | 38.1 |  |  | 61 |  | 60.0 |  |  |
| ≥65year |  | 279 |  | 61.9 |  |  | 47 |  | 40.0 |  |  |
| Gender |  |  |  |  |  |  |  |  |  |  |  |
| Female |  | 208 |  | 46.1 |  |  | 55 |  | 48.9 |  |  |
| Male |  | 243 |  | 53.9 |  |  | 53 |  | 51.1 |  |  |
| pT |  |  |  |  |  |  |  |  |  |  |  |
| T1 |  | 12 |  | 2.7 |  |  | 2 |  | 11.4 |  |  |
| T2 |  | 79 |  | 17.5 |  |  | 12 |  | 9.3 |  |  |
| T3 |  | 308 |  | 68.3 |  |  | 39 |  | 32.1 |  |  |
| T4 |  | 52 |  | 11.5 |  |  | 55 |  | 47.2 |  |  |
| pN |  |  |  |  |  |  |  |  |  |  |  |
| N0 |  | 266 |  | 59.0 |  |  | 76 |  | 69.0 |  |  |
| N1 |  | 105 |  | 23.3 |  |  | 24 |  | 20.7 |  |  |
| N2 |  | 80 |  | 17.7 |  |  | 8 |  | 10.3 |  |  |
| N3 |  | 0 |  | 0.0 |  |  | 0 |  | 0.0 |  |  |
| Metastasis |  |  |  |  |  |  |  |  |  |  |  |
| No |  | 335 |  | 74.3 |  |  | 106 |  | 92.9 |  |  |
| Yes |  | 116 |  | 25.7 |  |  | 2 |  | 7.1 |  |  |
| TNM |  |  |  |  |  |  |  |  |  |  |  |
| I |  | 78 |  | 17.3 |  |  | 2 |  | 10.9 |  |  |
| II |  | 176 |  | 39.0 |  |  | 20 |  | 32.1 |  |  |
| III |  | 134 |  | 29.7 |  |  | 84 |  | 42.4 |  |  |
| IV |  | 63 |  | 14.0 |  |  | 2 |  | 14.6 |  |  |
| Patient characteristics of TCGA were retrieved from <http://www.cbioportal.org> at April. 18th, 2020;  YJSHC means the Yijishan Hospital cohort; TCGA means the Cancer Genome Atlas cohort. | | | | | | | | | | | |
