# Supplementary material for: CXCL11 Correlates With Antitumor Immunity and an Improved Prognosis in Colon Cancer
Source: Front Cell Dev Biol. 2021 Mar 11;9:646252. doi: 10.3389/fcell.2021.646252 (PMC7991085; doi:10.3389/fcell.2021.646252)
Supplement: Supplementary Table 3 — Immunohistochemistry (IHC) antibodies. [file Table_3.DOCX]

Supplementary Material

| **Supplementary Table 3. Immunohistochemical (IHC) antibodies**. | | | | | | | |
| --- | --- | --- | --- | --- | --- | --- | --- |
| **No.** | **Antibody name** | **Description** | **Reactivity** | **Company** | **Product No.** | **Dilut** | **Identical cells** |
| 1 | Anti-CXCL11 antibody | Rabbit polyclonal | Human CXCL11 | Abcam | Ab9955 | 1:100 | CXCL11^+^ cells |
| 2 | Anti-PDL1  antibody | Rabbit monoclonal | Human PDL1 | Abcam | Ab205921 | 2 µg/ml | PDL1^+^ cells |
| 3 | Anti-CD8a  antibody | Rabbit monoclonal | Human CD8a | Abcam | Ab108343 | 1:100 | CD8^+^ T cells |
| 4 | Anti-NCAM antibody | Mouse monoclonal | Human CD56 | Abcam | ab9018 | 1:50 | CD56^+^ NK cells  (natural killer cells） |
|  |  |  |  |  |  |  |  |
