# Supplementary material for: CXCL11 Correlates With Antitumor Immunity and an Improved Prognosis in Colon Cancer
Source: Front Cell Dev Biol. 2021 Mar 11;9:646252. doi: 10.3389/fcell.2021.646252 (PMC7991085; doi:10.3389/fcell.2021.646252)
Supplement: Supplementary file 6 [file Table_4.DOCX]

Supplementary Material

**Supplementary Figure Legends**

**Supplementary Figure 1.** Tumor infiltrating lymphocytes (TILs) regulated by CXCL11 across all TCGA tumors. **(A)** The infiltration of various TILs between high and low CXCL11 mRNA expression groups across all TCGA tumors in TISIDB. Act CD8 = activated CD8^+^ T cells; Tcm CD8 = central memory CD8^+^ T cells; Tem CD8 = effector memory CD8^+^ T cells; Act CD4; Tcm CD4 = activated CD4^+^ T cells; Tem CD4 = effector memory CD4^+^ T cells; Tfh =follicular helper T cells; Tgd = gamma delta T cells; Th1 = T helper 1 cells; Th17 = T helper 17 cells; Th2 = T helper 2 cells; Treg = regulatory T cells; Act B = activated B cells; Imm B = immature B cells; Mem B = memory B cells; NK = natural killer cells; CD56bright = CD56^+^ cells；CD56dim = CD56^-^ cells; MDSC; NKT = natural killer T cells; Act DC = activated dendritic cells; pDC = plasmocytoid dendritic cells; iDC = immature dendritic cells; Macrophage = macrophages; Eosinophil = eosinophils; Mast = mast cells; Monocyte = monocytes; Neutrophil = neutrophils. ACC = adrenocortical carcinoma; BLCA = bladder urothelial carcinoma; BRCA = breast carcinoma; CESC = cervical squamous carcinoma; CHOL = cholangiocarcinoma; COAD = colon adenocarcinoma; ESCA = esophageal carcinoma; GBM = glioblastoma multiforme; HNSC = head and neck squamous cell carcinoma; KICH= Kidney Chromophobe; KIRC = Kidney renal clear cell carcinoma; KIRP = Kidney renal papillary cell carcinoma; LGG = lower grade glioma; LIHC = liver hepatocellular carcinoma; LUAD = lung adenocarcinoma; LUSC = lung squamous cell carcinoma; MESO = mesothelioma; OV = ovarian serous adenocarcinoma; PAAD = pancreatic ductal adenocarcinoma; PCPG = paraganglioma & pheochromocytoma; PRAD = prostate adenocarcinoma; READ = Rectum adenocarcinoma; SARC = sarcoma; SKCM = skin cutaneous melanoma; STAD; TGCT = testicular germ cell cancer; THCA = thyroid papillary carcinoma; UCEC = uterine corpus endometrioid carcinoma; UCS = uterine corpus squamous carcinoma; UVM = uveal melanoma.

**Supplementary Figure 2.** UMAP plots showing expression levels of selected genes in different clusters of GSE146771. **(A)** UMAP plots showing expression levels of CXCL11 in different clusters. **(B)** UMAP plots showing expression levels of PD-L1 in different clusters. **(C)** UMAP plots showing different clusters.
